# A Machine Learning Approach for Predicting Biochemical Outcome After PSMA-PET–Guided Salvage Radiotherapy in Recurrent Prostate Cancer After Radical Prostatectomy: Retrospective Study

**DOI:** 10.2196/60323

**Published:** 2024-09-20

**Authors:** Ali Janbain, Andrea Farolfi, Armelle Guenegou-Arnoux, Louis Romengas, Sophia Scharl, Stefano Fanti, Francesca Serani, Jan C Peeken, Sandrine Katsahian, Iosif Strouthos, Konstantinos Ferentinos, Stefan A Koerber, Marco E Vogel, Stephanie E Combs, Alexis Vrachimis, Alessio Giuseppe Morganti, Simon KB Spohn, Anca-Ligia Grosu, Francesco Ceci, Christoph Henkenberens, Stephanie GC Kroeze, Matthias Guckenberger, Claus Belka, Peter Bartenstein, George Hruby, Louise Emmett, Ali Afshar Omerieh, Nina-Sophie Schmidt-Hegemann, Lucas Mose, Daniel M Aebersold, Constantinos Zamboglou, Thomas Wiegel, Mohamed Shelan

**Affiliations:** 1 European Hospital Georges-Pompidou. Clinical research unit, INSERM Clinical Investigation Center. Paris Cité University Paris France; 2 Division of Nuclear Medicine IRCCS Azienda Ospedaliero-Universitaria di Bologna Bologna Italy; 3 Department of Radiation Oncology University of Ulm Ulm Germany; 4 Department of Radiation Oncology Klinikum rechts der Isar Technical University of Munich (TUM) Munich Germany; 5 Department of Radiation Oncology German Oncology Center University Hospital of the European University Limassol Cyprus; 6 Department of Radiation Oncology Heidelberg University Hospital Heidelberg Germany; 7 Division of Radiation Oncology IRCCS Azienda Ospedaliero-Universitaria di Bologna Bologna Italy; 8 Department of Radiation Oncology Medical Center–University of Freiburg Faculty of Medicine, University of Freiburg Freiburg Germany; 9 Division of Nuclear Medicine IEO European Institute of Oncology IRCCS Milan Italy; 10 Department of Radiotherapy and Special Oncology Medical School Hannover Hannover Germany; 11 Department of Radiation Oncology University Hospital Zürich University of Zurich Zurich Switzerland; 12 Department of Radiation Oncology University Hospital, LMU Munich Munich Germany; 13 Department of Nuclear Medicine University Hospital, LMU Munich Munich Germany; 14 Department of Radiation Oncology Royal North Shore Hospital–University of Sydney Sydney Australia; 15 Department of Theranostics and Nuclear Medicine St Vincent’s Hospital Sydney Sydney Australia; 16 Department of Nuclear Medicine Inselspital, Bern University Hospital University of Bern Bern Switzerland; 17 Department of Radiation Oncology KSA-KSB Cantonal Hospital Aarau Aarau Switzerland; 18 Department of Radiation Oncology Inselspital, Bern University Hospital University of Bern Bern Switzerland

**Keywords:** cancer, oncologist, oncologist, metastases, prostate, prostate cancer, prostatectomy, salvage radiotherapy, PSMA-PET, prostate-specific membrane antigen–positron emission tomography, prostate-specific membrane antigen, PET, positron emission tomography, radiotherapy, radiology, radiography, machine learning, ML, artificial intelligence, AI, algorithm, algorithms, predictive model, predictive models, predictive analytics, predictive system, practical model, practical models, deep learning

## Abstract

**Background:**

Salvage radiation therapy (sRT) is often the sole curative option in patients with biochemical recurrence after radical prostatectomy. After sRT, we developed and validated a nomogram to predict freedom from biochemical failure.

**Objective:**

This study aims to evaluate prostate-specific membrane antigen–positron emission tomography (PSMA-PET)–based sRT efficacy for postprostatectomy prostate-specific antigen (PSA) persistence or recurrence. Objectives include developing a random survival forest (RSF) model for predicting biochemical failure, comparing it with a Cox model, and assessing predictive accuracy over time. Multinational cohort data will validate the model’s performance, aiming to improve clinical management of recurrent prostate cancer.

**Methods:**

This multicenter retrospective study collected data from 13 medical facilities across 5 countries: Germany, Cyprus, Australia, Italy, and Switzerland. A total of 1029 patients who underwent sRT following PSMA-PET–based assessment for PSA persistence or recurrence were included. Patients were treated between July 2013 and June 2020, with clinical decisions guided by PSMA-PET results and contemporary standards. The primary end point was freedom from biochemical failure, defined as 2 consecutive PSA rises >0.2 ng/mL after treatment. Data were divided into training (708 patients), testing (271 patients), and external validation (50 patients) sets for machine learning algorithm development and validation. RSF models were used, with 1000 trees per model, optimizing predictive performance using the Harrell concordance index and Brier score. Statistical analysis used R Statistical Software (R Foundation for Statistical Computing), and ethical approval was obtained from participating institutions.

**Results:**

Baseline characteristics of 1029 patients undergoing sRT PSMA-PET–based assessment were analyzed. The median age at sRT was 70 (IQR 64-74) years. PSMA-PET scans revealed local recurrences in 43.9% (430/979) and nodal recurrences in 27.2% (266/979) of patients. Treatment included dose-escalated sRT to pelvic lymphatics in 35.6% (349/979) of cases. The external outlier validation set showed distinct features, including higher rates of positive lymph nodes (47/50, 94% vs 266/979, 27.2% in the learning cohort) and lower delivered sRT doses (<66 Gy in 57/979, 5.8% vs 46/50, 92% of patients; *P*<.001). The RSF model, validated internally and externally, demonstrated robust predictive performance (Harrell C-index range: 0.54-0.91) across training and validation datasets, outperforming a previously published nomogram.

**Conclusions:**

The developed RSF model demonstrates enhanced predictive accuracy, potentially improving patient outcomes and assisting clinicians in making treatment decisions.

## Introduction

Prostate-specific antigen (PSA) is a protein produced by the prostate gland, and its levels in the blood are commonly used as a marker in the assessment of prostate health. PSA levels are measured using an immunoassay, and elevated levels can be indicative of prostate conditions including benign prostatic hyperplasia or prostate cancer. Biochemical recurrence (BR) refers to the increase in PSA levels after treatment; this occurs in approximately 15% to 25% of patients following radical prostatectomy (RP) for prostate cancer [1]. While BR does not invariably lead to metastatic progression and death, the risk significantly increases [2]. Salvage radiation therapy (sRT) offers these patients with localized disease a second chance at a cure [2-4]. Historically, prognostic nomograms by Stephenson et al [5] and Tendulkar et al [6] provided valuable insights into predicting outcomes after sRT. The Stephenson nomogram was developed on a cohort of patients with a median PSA value of 1.1 (IQR 0.6-2.2) ng/mL. In contrast, the Tendulkar nomogram included patients managed with ultrasensitive PSA assays, with a median pre-sRT PSA of 0.5 (IQR 0.3-1.1) ng/mL.

However, recent advances in imaging have rendered traditional recurrence prediction models obsolete. Prostate-specific membrane antigen–positron emission tomography (PSMA-PET) is a diagnostic tool that uses PSMA ligands to identify prostate cancer. PSMA, a surface protein highly expressed in prostate cancer cells, enables PSMA-PET to achieve exceptional sensitivity and specificity in detecting cancer recurrence [7,8]. This high precision allows for more tailored and effective radiotherapy planning. Both retrospective and prospective studies have demonstrated that integrating PSMA-PET data before sRT modifies the treatment strategy in approximately 30% to 50% of cases. [9,10]. This effect is evident even in patients undergoing early sRT with PSA levels below 0.5 ng/mL, as this group’s detection rate is approximately 50% [10,11].

Machine learning (ML) algorithms are increasingly used to create prediction tools because they can swiftly process vast datasets. They have been demonstrated to outperform clinical experts in estimating patient survival in a cohort of patients with lung cancer [12]. Comparisons of outcome prediction models in other entities provided evidence that the reliability of ML-based tools may be superior to those generated by traditional nomograms [13,14]. Given these advancements, new risk models are needed to predict sRT outcomes in the PSMA-PET era.

In previous work from our group, we developed a nomogram to predict outcomes in patients with prostate cancer undergoing sRT after RP [15]. In this study, we present a ML-based random survival forest (RSF) model for risk prediction, using a substantial international dataset of patients who underwent PSMA-PET staging before sRT. We compared the prediction accuracy with our previously published nomogram. This study represents the first prediction tool for PSMA-PET–staged patients using a ML-based method derived from a large international patient cohort.

## Methods

### Source of Data

Data for this study were contributed by 13 medical facilities across 5 different countries: Germany (n=6), Cyprus (n=1), Australia (n=3), Italy (n=1), and Switzerland (n=2). Each facility contributed between 20 and 175 patients to the cohort (for more details, see Multimedia Appendix 1). The participation of these institutions in this multicenter study was approved by the respective ethics committees. Reporting adhered to the STROBE (Strengthening the Reporting of Observational Studies in Epidemiology) reporting guidelines (Multimedia Appendix 1). All ethics committees of the included institutions approved this study.

### Participants

Patients who underwent open or laparoscopic RP and received PSMA-PET–based sRT for PSA persistence or recurrence (PSA levels ≥0.1 ng/ml postprostatectomy) were included in this study. Written informed consent was not required due to the retrospective nature of the investigation and by review board guidelines. Exclusion criteria involved distant metastases on PSMA-PET or computed tomography scan and initiation of androgen deprivation therapy (ADT) before PSMA-PET or computed tomography scan. A total of 1221 patients met the inclusion criteria and underwent sRT between July 1, 2013, and June 30, 2020. Out of these, 192 individuals were excluded: 141 individuals had insufficient clinical data, 47 individuals had no prostatic fossa in the sRT field, and 4 individuals had PSMA-PET–positive lesions outside the sRT field.

Consequently, 1029 patients with complete data participated in developing and validating the ML algorithm. A total of 50 patients’ data were used for external validation, 708 patients’ data were used for training, and 271 patients’ data were used for testing.

No formal sample size was elaborated. All patients with inclusion criteria were supposed to be eligible for the analysis, and the number of participants was deemed relevant to developing ML algorithms.

### Treatment and Follow-Up

Treating clinicians made clinical choices based on PSMA-PET results and current standards of care. The institutional clinical practice involved intensity-modulated, image-guided sRT to the prostatic fossa, occasionally with a concurrent integrated boost to local recurrence. Additional treatments, such as elective pelvic lymphatic radiation and ADT, were administered based on patient risk characteristics. Follow-up evaluations adhered to institutional clinical practices including periodic serum PSA testing and restaging for BR. BR was defined as 2 consecutive rising PSA values >0.2 ng/mL after treatment.

### Predictors

Predictors were strictly the same as in the previous work from our group [15]. They included the International Society of Urological Pathology grade of the surgical specimen, pathological T stage (pT stage), resection status, PSA serum values before sRT, ADT use, dose in the prostate, persistence of PSA levels after surgery, and presence of pelvic lymph nodes or local recurrence before sRT. Based on clinical expertise, some variables with limited predictive value in previous studies were excluded from the analysis [6,16].

### Statistical Analysis—Model Development and Validation

We used the RSF classifier for survival analysis, an extension of the random forests ML algorithms in a context of right-censored survival data, based on prior research demonstrating its efficacy in predicting freedom from biochemical failure (FFBF), defined as 2 consecutive PSA rises >0.2 ng/mL after treatment, after sRT [17]. We first separated the dataset into 2 parts: an external outlier validation dataset and a learning dataset.

The outlier validation dataset consisted of 50 patients from the most dissimilar center, which was selected based on a principal component analysis that excluded the center variable (see our previous published work [15]). This ensured that the validation dataset represented a more diverse range of patients than the learning dataset.

The remaining patients (979 patients in total) were used to develop 900 models. Indeed, we selected 30 seeds at random between 1 and 10,000 with uniform distribution. The seeds ensure different random splits of the data, while the uniform distribution avoids bias by giving each seed an equal chance of being chosen. For each seed, to provide an accurate assessment of RSF internal validity, we divided 30 times the learning dataset into training and internal validation datasets (ratio 75:25) with stratified random sampling for stratification factors (see eTable 4 in Supplement 1 in Zamboglou et al [15]), allowing the use of common attributes in the data to form strata before sampling, resulting in a more representative and general sample.

Each model resulted from an RSF that was grown using 1000 trees. Simultaneous optimizations of the number of trees in the forest and the number of predictors available to be selected from at each split were obtained by a grid search (100, 500, and 1000 for the number of trees; 1 to 8 for the number of predictors) with 10-fold cross-validation on the training dataset. The splitting rule was based on the logrank test. A random selection of split points is considered for each predictor.

Several metrics served to evaluate the predictive performances of each model. First, we used the Harrell concordance index (C-index) [18]. The higher the C-index, the better the discriminatory power of the model. The Harrell C-index was further classified according to the Altman Strength of Agreement [19]. Separate boxplots graphically represented the distribution of the predictive performances, in each dataset. We defined the model having the highest Harrell C-index on the internal validation dataset as our best RSF model. The corresponding seed for randomness is given.

Second, we added the Brier score, which served as a measure of both discrimination and calibration [20]. The lower the Brier score, the higher the predictive quality of the model. Minimal and maximal values (ie, range values) of the Harrell C-index and Brier score in the training, internal validation, and external outlier datasets were given separately.

The importance and relative importance values of the predictors were calculated. The relative importance provides a normalized measure, allowing for a comparison between predictors. The higher the value, the greater the importance of variables in the outcome prediction.

The Harrell C-index and Brier score of our best RSF model were measured at each time point between 12 and 85 months, with an interval of 1 month, in the training, internal validation, and external outlier validation datasets. To compare the prediction accuracy with our Cox proportional hazard model, previously published as a nomogram, we applied the latter at each time point, too. Results were displayed graphically, presenting the Harrell C-index and Brier score from our best RSF model and previous Cox proportional hazard model as a function of time. Minimal and maximal values (ie, range values) of the Harrell C-index and Brier score in the training, internal validation, and external outlier datasets were given separately.

All statistical analysis was conducted using R Statistical Software (version 4.2.1; R Foundation for Statistical Computing). Descriptive statistics are given by either range, median (IQR), or number (percentage in %), according to variable nature. Stratified random sampling was performed using the *Splitstackshape* package (version 1.4.8). The Fisher exact or chi-square test was used to compare clinical and treatment characteristics between different subdatasets. We used the *randomForestSRC* package (version 3.2.1) for RFS model training and *SurvMetrics* (version 0.5.0) for the Harrell C-index and Brier score. The importance and relative importance values of the predictors in the RSF were calculated using the *VIMP* function [21]. A 2-sided *P* value of <.05 was considered as the significance level.

### Ethics Considerations

This study adhered to ethical standards across all recruiting centers, with ethical approval obtained from each institution involved. Given the retrospective nature of the study, informed consent was waived, as is permitted for studies involving secondary analysis of existing data. The primary data collection was conducted under the appropriate ethical guidelines, with the original informed consent covering the use of data for secondary analysis without requiring additional consent. To ensure privacy and confidentiality, all study data were deidentified, maintaining the anonymity of participants. No compensation was provided to participants in this study, reflecting the nature of the research and ensuring transparency in the process. The file number for ethical approval from Bern University Hospital is BE 2021-02294.

## Results

### Baseline Patient and Treatment Characteristics

In this study, we adopted the same formulation as previously published [15]. We analyzed the baseline patients and treatment characteristics of the entire cohort, which consisted of 1029 patients with a median age at sRT of 70 (IQR 64-74) years. For that publication, the cohort was already divided into a training set (n=708), an internal validation set (n=271), and an external outlier validation dataset (n=50), and these groups are summarized in Table 1.

Within the learning cohort (comprising the training and internal validation sets; n=979), most patients (n=610, 62.3%) had PSA serum values of 0.5 ng/mL or less before sRT. Locally recurrent disease detected by PET scan was present in 43.9% (n=430) of patients, while 27.2% (n=266) of patients had at least 1 positive pelvic lymph node on PET scan. Among the patients, 32.2% (n=315) of patients received ADT without any escalation of systemic therapy beyond ADT. The most commonly administered equivalent dose of 2 Gy per fraction (EQD2, α/β=1.6 Gy) to the prostatic fossa or locally recurrent disease was 66 to 70 Gy (n=547, 55.9% of patients).

PSMA-PET scans conducted before sRT revealed local recurrences in 43.9% (n=430) of patients and nodal recurrences in 27.2% (n=266) of patients. sRT to elective pelvic lymphatics was administered to 35.6% (n=349) of patients. All pelvic lymph node PETs received dose-escalated sRT; the most frequent dose (149/317, 56%) was 50 to 60 Gy (EQD2, α/β=1.6 Gy).

No significant difference in clinical and treatment characteristics was observed between the patients in the training and the internal validation cohorts (all *P*>.05; Table 2). However, the external outlier cohort exhibited distinct features, with no patients having negative PSMA-PET scans, significantly higher rates of complete resection (44/50, 88% vs 629/979, 64.2% of patients; *P*=.001), and a significantly greater proportion of patients with positive pelvic lymph nodes (47/50, 94% vs 266/979, 27.2% of patients; *P*<.001) as compared to the learning cohort (Table 3). Furthermore, the delivered dose to the prostatic fossa was significantly lower for the patients in the external outlier cohort than in the learning cohort (57/979, 5.8% vs 46/50, 92% of patients with a dose less than 66 Gy; *P*<.001).

**Table 1:** Baseline treatment characteristics among training set, internal validation set, and external outlier validation set.

Characteristic			Total cohort (n=1029)		Training dataset (n=708)		Internal validation dataset (n=271)		External outlier validation dataset (n=50)
Age at sRT^a^ (years), median (IQR)			70 (64-74)		70 (64-74)		69 (63-74)		72.5 (68-76)
**pT stage^b^, n (%)**									
	2	460 (44.7)		310 (43.8)		122 (45)		28 (56)	
	3a	327 (31.8)		230 (32.5)		86 (31.7)		11 (22)	
	3b	235 (22.8)		163 (23)		61 (22.5)		11 (22)	
	4	7 (0.7)		5 (0.7)		2 (0.7)		0 (0)	
**R status^c^ in surgery, n (%)**									
	RO	673 (65.4)		448 (63.3)		181 (66.8)		44 (88)	
	R1	327 (31.8)		244 (34.5)		77 (28.4)		6 (12)	
	R2	3 (0.3)		1 (0.1)		2 (0.7)		0 (0)	
	Rx	26 (2.5)		15 (2.1)		11 (4.1)		0 (0)	
**ISUP^d^ grade in surgery, n (%)**									
	1+2	371 (36.1)		254 (35.9)		101 (37.3)		16 (32)	
	3	324 (31.5)		226 (31.9)		84 (31)		14 (28)	
	4	156 (15.2)		102 (14.4)		44 (16.2)		10 (20)	
	5	178 (17.3)		126 (17.8)		42 (15.5)		10 (20)	
**PSA^e^ persistence after surgery, n (%)**									
	No	750 (72.9)		511 (72.2)		197 (72.7)		42 (84)	
	Yes	279 (27.1)		197 (27.8)		74 (27.3)		8 (16)	
**PSA (ng/mL) before sRT, n (%)**									
	0.01-0.2	246 (23.9)		178 (25.1)		63 (23.3)		5 (10)	
	>0.2-0.5	385 (37.4)		258 (36.4)		111 (41)		16 (32)	
	>0.5-1	172 (16.7)		122 (17.2)		41 (15.1)		9 (18)	
	>1	226 (22)		150 (21.2)		56 (20.7)		20 (40)	
**Local recurrence after PSMA-PET^f^, n (%)**									
	No	592 (57.5)		396 (55.9)		153 (56.5)		43 (86)	
	Yes	437 (42.5)		312 (44.1)		118 (43.5)		7 (14)	
**Pelvic lymph nodes after PSMA-PET, n (%)**									
	No	716 (69.6)		507 (71.6)		206 (76)		3 (6)	
	Yes	313 (30.4)		201 (28.4)		65 (24)		47 (94)	
**Dose to the prostatic fossa (Gy^g^), n (%)**									
	<66	103 (10)		47 (6.6)		10 (3.7)		46 (92)	
	66-70	551 (53.6)		390 (55.1)		157 (57.9)		4 (8)	
	>70	375 (36.4)		271 (38.3)		104 (38.4)		0 (0)	
**sRT to elective pelvic lymphatics, n (%)**									
	No	633 (61.6)		455 (64.4)		174 (64.2)		4 (8)	
	Yes	395 (38.4)		252 (35.6)		97 (35.8)		46 (92)	
**Dose to elective pelvic lymphatics (Gy), n (%)**									
	<50	312 (30.3)		197 (27.8)		71 (26.2)		44 (100)	
	>50	47 (4.6)		34 (4.80)		13 (4.79)		0 (0)	
	Unknown	36 (3.5)		21 (3)		13 (4.8)		2 (4)	
**Irradiation to positive pelvic LN^h^, n (%)**									
	No	712 (69.2)		505 (71.3)		204 (75.3)		3 (6)	
	Yes	317 (30.8)		203 (28.7)		67 (24.7)		47 (94)	
**Dose to positive pelvic LNs (Gy), n (%)**									
	<50	15 (1.5)		11 (1.6)		4 (1.5)		0 (0)	
	50-60	149 (13.5)		113 (16)		36 (13.3)		0 (0)	
	>60	128 (12.4)		63 (8.9)		20 (7.4)		45 (90)	
	Unknown	25 (2.4)		16 (2.3)		7 (2.6)		2 (4)	
**ADT^i^, n (%)**									
	No	704 (68.4)		475 (67.1)		189 (69.7)		40 (80)	
	Yes	325 (31.6)		233 (32.9)		82 (30.3)		10 (20)	
**Duration of ADT admission (months), n (%)**									
	<6	65 (23.1)		50 (24.4)		15 (22.7)		0 (0)	
	6-12	110 (39.2)		79 (38.5)		24 (36.4)		7 (70)	
	>12-24	57 (20.3)		39 (19.0)		18 (27.3)		0 (0)	
	>24	49 (17.4)		37 (18.1)		9 (13.6)		3 (30)	
	Unknown	44 (4.3)		28 (4.0)		16 (5.9)		0 (0)	

^a^sRT: salvage radiation therapy.

^b^pT stage: pathological T stage.

^c^R status: residual disease status.

^d^ISUP: International Society of Urological Pathology.

^e^PSA: prostate-specific antigen.

^f^PSMA-PET: prostate-specific membrane antigen–positron emission tomography.

^g^Gy: gray (a unit of radiation dose).

^h^LN: lymph node.

^i^ADT: androgen deprivation therapy.

**Table 2:** Comparison between training cohort and internal outlier validation datasets (*P* value based on Fisher exact or chi-square test).

Covariate, n (%)		Training dataset (n=708), n (%)	Internal validation dataset (n=271), n (%)	*P* value	
**pT stage^a^**					.94
	pT2	310 (43.8)	122 (45)		
	pT3a	230 (32.5)	86 (31.7)		
	pT3b+pT4	168 (23.7)	63 (23.3)		
**R status^b^**					.31
	R0	448 (63.3)	181 (66.8)		
	R1/2+Rx	260 (36.7)	90 (33.2)		
**ISUP^c^ grade**					.69
	1+2	254 (35.9)	101 (37.3)		
	3+4	328 (46.3)	128 (47.2)		
	5	126 (17.8)	42 (15.5)		
**Pelvic lymph nodes on PET^d^**					.17
	No	507 (71.6)	206 (76)		
	Yes	201 (28.4)	65 (24)		
**PSA^e^ prior to sRT^f^**					.45
	<0.5 ng/mL	436 (61.6)	174 (64.2)		
	>0.5 ng/mL	272 (38.4)	97 (35.8)		
**sRT dose to the prostatic fossa^f^**					.20
	<66 Gy^g^	47 (6.6)	10 (3.69%)		
	66-70 Gy vs <66 Gy	390 (55.1)	157 (57.9)		
	>70 Gy	271 (38.3)	104 (38.4)		
**ADT^i^**					.43
	No	475 (67.1)	189 (69.7)		
	Yes	233 (32.9)	82 (30.3)		

^a^pT stage: pathological T stage.

^b^R status: residual disease status.

^c^ISUP: International Society of Urological Pathology.

^d^PET: positron emission tomography.

^e^PSA: prostate-specific antigen.

^f^sRT: salvage radiation therapy.

^g^Gy: gray (a unit of radiation dose).

^i^ADT: androgen deprivation therapy.

**Table 3:** Comparison between learning cohort (training+internal validation cohort) and external outlier cohort (*P* value based on Fisher exact or chi-square test).

Covariate, n (%)			Learning (n=979), n (%)		External (n=50), n (%)		*P* value	
**pT stage^a^**								.21
	pT2	432 (44.1)		28 (56)				
	pT3a	316 (32.3)		11 (22)				
	pT3b+pT4	231 (23.6)		11 (22)				
**R status^b^**								.001
	R0	629 (64.2)		44 (88)				
	R1/2+Rx	350 (35.8)		6 (12)				
**ISUP^c^ grade**								.79
	1+2	355 (36.3)		16 (32)				
	3+4	456 (46.6)		24 (48)				
	5	168 (17.2)		10 (20)				
**Pelvic lymph nodes on PET^d^**								<.001
	No	713 (72.9)		3 (6)				
	Yes	266 (27.2)		47 (94)				
**PSA^e^ before sRT^f^**								.004
	<0.5 ng/ml	610 (62.3)		21 (42)				
	>0.5 ng/ml	369 (37.7)		29 (58)				
**sRT dose to the prostatic fossa**								<.001
	<66 Gy^g^	57 (5.8)		46 (92)				
	66-70 Gy vs <66 Gy	547 (55.9)		4 (8)				
	>70 Gy	375 (38.3)		0 (0)				
**ADT^g^**								.07
	No	664 (67.8)		40 (80)				
	Yes	315 (32.2)		10 (20)				

^a^pT stage: pathological T stage.

^b^R status: residual disease status.

^c^ISUP: International Society of Urological Pathology.

^d^PET: positron emission tomography.

^e^PSA: prostate-specific antigen.

^f^sRT: salvage radiation therapy.

^g^Gy: gray (a unit of radiation dose).

^h^ADT: androgen deprivation therapy.

Among the patients with positive lymph nodes detected on PET scans (n=349), 52.4% (n=183) of patients received ADT, whereas 47.6% (n=166) of patients did not. No significant difference was observed in the distribution of the International Society of Urological Pathology (ISUP) grade and pT stage (all *P*>.05; Table 3).

### Model Development and Validation

All training subsets comprised 708 patients while corresponding internal validation sets contained the 271 remaining patients.

**Figure 1:**
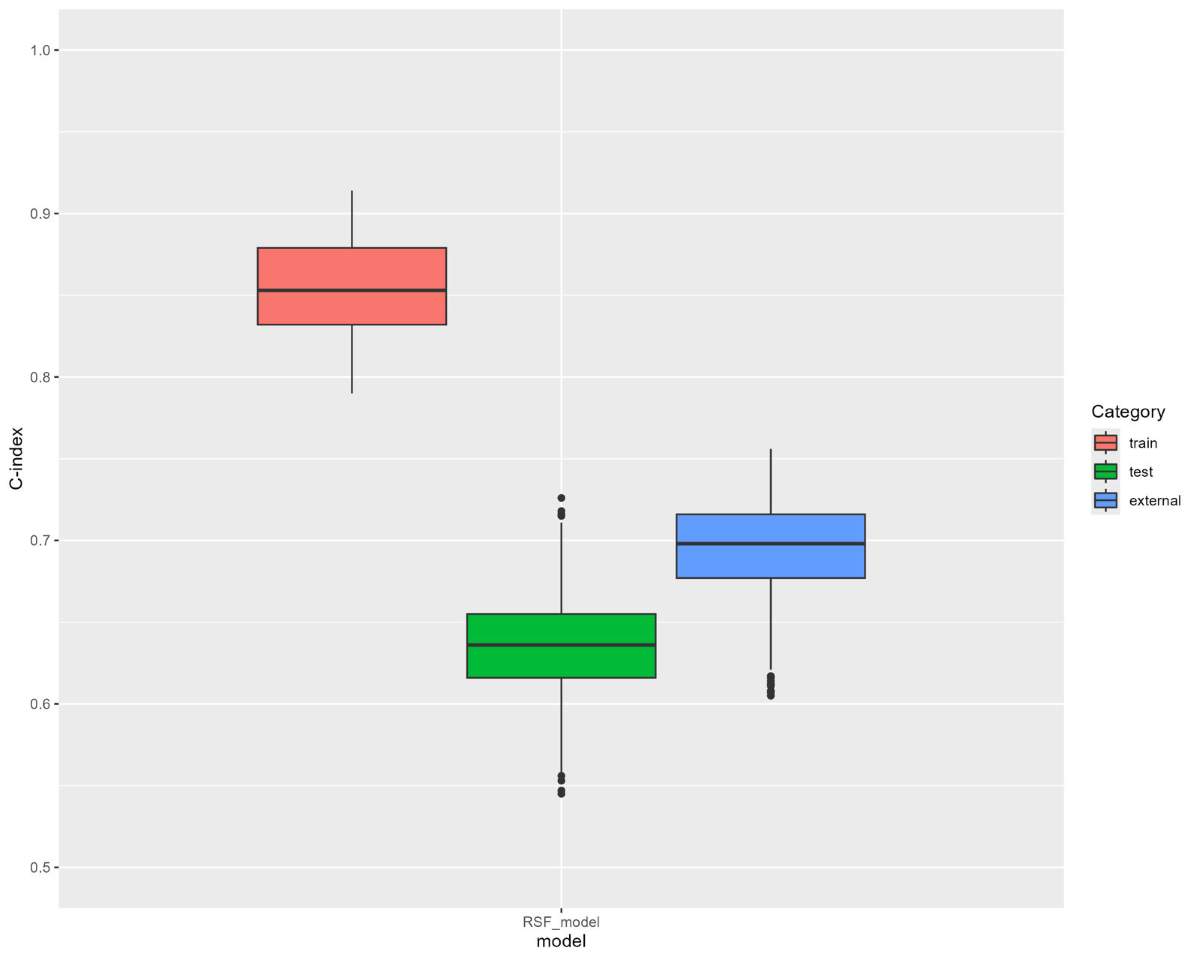
Harrell C-indexes of the 900 RSF models in the training, internal validation, and external validation datasets. C-index: concordance index. RSF: random survival forest.

Figure 1 summarizes the performance obtained from the 900 developed RSF models after training and further application to the internal and external outlier validation datasets. The Harrell C-index values of the training datasets showed good concordances ranging from 0.79 to 0.91. The internal validation dataset showed moderate to good concordances ranging from 0.54 to 0.73, while the external outlier validation dataset, showed good concordances ranging from 0.60 to 0.76.

Our best model (the highest Harrell C-index on the internal validation dataset) was the one at the 15th iteration with the seed being 7332. Its Harrell C-index values were 0.79, 0.72, and 0.69 in the training, internal validation, and external outlier validation datasets, respectively. The corresponding training and internal validation recorded 200 (28.2%) out of 708 and 77 (28.4%) out of 271 patients with cancer relapse, respectively.

Correspondingly, Brier score results ranged from 0.12 to 0.15, from 0.10 to 0.20, and from 0.12 to 0.16 in the training, internal validation, and external outlier validation datasets, respectively. Brier scores related to our best model equaled 0.13, 0.14, and 0.14, respectively.

Table 4 presents the importance and relative importance values of the predictors in our best RSF model. The predictors with the highest importance values were PSA before sRT and pelvic nodal recurrence. Conversely, the predictor with the lowest importance value was PSA persistence.

In training, internal validation, and external outlier validation datasets, our best RSF model exhibited higher Harrell C-indexes (0.79, 0.72, and 0.69) than our nomogram previously published (0.68, 0.72, and 0.67, respectively). Our best RSF model showed higher Brier scores but more stable results across the datasets than the model for our nomogram (best RSF=0.13, 0,14, 0,14 vs Cox=0.12, 0,13, 0,15 for training, internal validation, and external outlier validation datasets, respectively).

**Figure 2:**
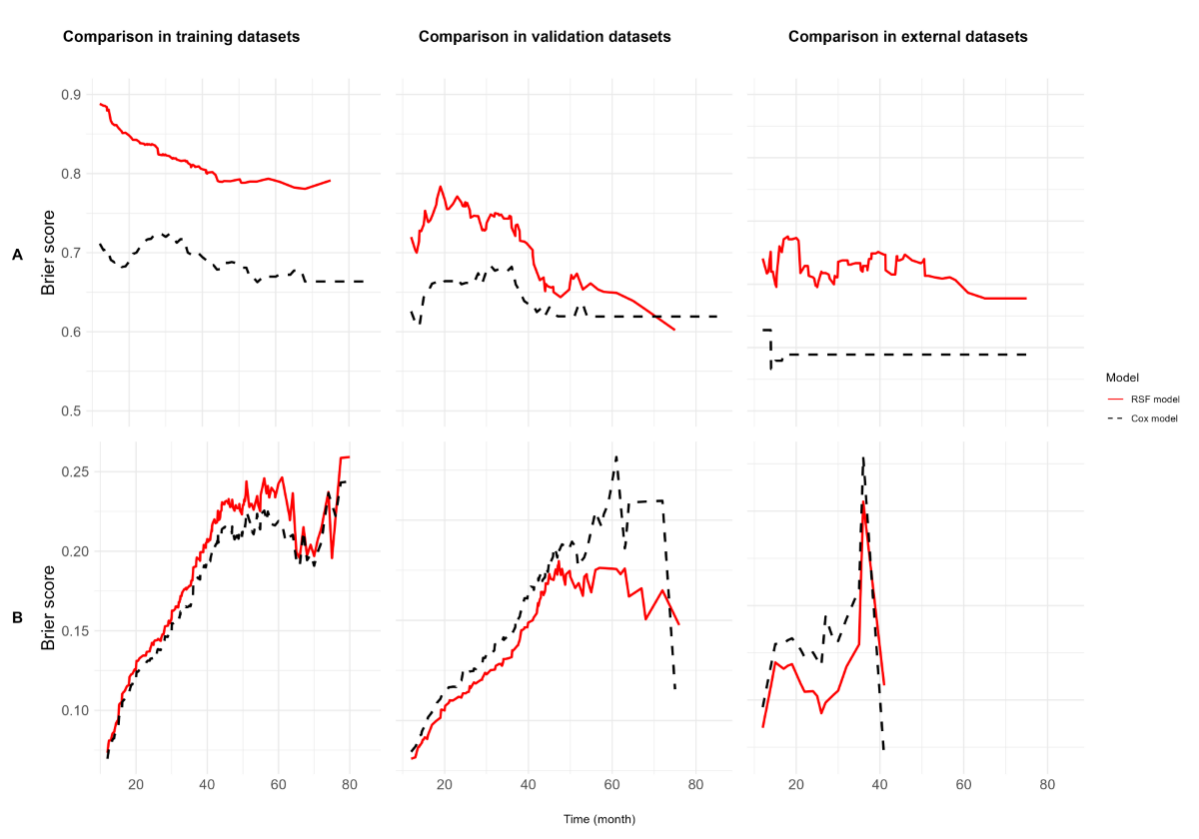
(A) Harrell C-indexes and (B) Brier scores of our best RSF model versus our Cox proportional hazard model previously published, in the training, internal validation, and external validation datasets over time (12-85 months interval, with a 1-month increment). RSF: random survival forest.

The Harrell C-indexes of our best RSF model compared to our nomogram previously published, when measured at time points of 12-85 months with an interval of 1 month in training, internal, and external outlier validation datasets, are shown in Figure 2A, while Figure 2B shows the Brier scores of our best RSF model and our nomogram previously published when measured at time points of 12-85 months with an interval of 1 month, according to the subdatasets.

**Table 4:** Predictor importance and relative importance in the RSF^a^ model.

Predictor	Importance	Relative importance
PSA^b^ prior sRT^c^ (ng/mL)	0.071	1
Pelvic nodal recurrence on PET^d^	0.065	0.920
pT status^e^	0.055	0.777
ISUP^f^ grade	0.050	0.705
Dose to prostatic fossa (Gy^g^)	0.029	0.415
ADT^h^	0.021	0.299
R status^i^	0.014	0.190
Pelvic local recurrence on PET	0.012	0.175
PSA persistence	–0.008	–0.116

^a^RSF: random survival forest.

^b^PSA: prostate-specific antigen.

^c^sRT: salvage radiation therapy.

^d^PET: positron emission tomography.

^e^pT status: pathological T status.

^f^ISUP: International Society of Urological Pathology.

^g^Gy: gray (a unit of radiation dose).

^h^ADT: androgen deprivation therapy.

^i^R status: residual disease status.

## Discussion

### Principal Findings

This study is the first study reporting an RSF model on prostate cancer patients across 5 countries undergoing PSMA-PET–based sRT. It presents a robust predictive performance (Harrel C-index 0.54-0.91) and outperforms the previously published nomogram.

### Comparison to Prior Work

The medical community is constantly striving to develop and refine predictive tools that can accurately identify the most effective care management options for patients. By doing so, health care providers can offer personalized care that maximizes patient outcomes while minimizing adverse reactions and being cost-effective. Nomograms were and are constantly used, and the great potential of an ML approach for dynamic prediction in medicine is now emerging [22]. In this context, using a large international dataset of patients who underwent PSMA-PET staging before sRT, this study aimed at developing a ML-based RSF model to predict FFBF and comparing the prediction performances with our previously published nomogram based on a Cox proportional hazards model [15].

Our best RSF model performed well after training (Harrell C-index=0.79). Furthermore, it showed good robustness and generalizability, maintaining good performances on the internal validation set (C-index=0.72) and the external outlier validation dataset (C-index=0.69). In all cases, our best RSF model outperformed the previously built nomogram on the same datasets (0.67, 0.71, and 0.66). Our previously published nomogram included 7 variables found to be statistically significant in our multivariable Cox proportional hazards regression analysis (pre-sRT PSA level, ISUP grade in surgery specimen, pT stage, surgical margins, ADT use, sRT dose to the fossa, and nodal recurrence detected on PSMA-PET scans) [15]. In addition to these 7 variables, our best RSF evaluated PSA persistence, based on the known literature of poor prognosis when this characteristic is present, and pelvic local recurrence on PSMA-PET scan, based on a recent paper showing that the presence of local recurrence was associated with favorable BR-free survival [23-25].

Out of the 9 variables, the one with the highest importance was the value of PSA before sRT, which was consequently associated with a relative importance of the model of 1. This result confirms what is known in the literature, and specifically, in a very recent paper studying a retrospective cohort of 25,551 patients over a period time of 30 years, it was found that performing sRT when PSA values fall above 0.25 ng/mL was associated with an increased all-cause mortality risk [24,26]. The second variable with the highest relative importance (0.92) for the model was the nodal recurrence detected on PSMA-PET scans. These data confirm the importance of performing a PSMA-PET in patients with BR, as well as the data found in the previous preliminary analysis [16,25].

On the other hand, the presence of pelvic local recurrence on the PSMA-PET scan had a relative importance of 0.175. Surprisingly, PSA persistence had a negative relative importance (–0.11). This statistical result suggests that randomly shuffling this variable helped the model perform slightly better, meaning that the variable might be adding confusion rather than helping with predictions. This needs to be further analyzed, as this would imply that PSA persistence after RP may have a negligible impact on the prediction of the outcome of sRT.

Yet, our findings align with the guidelines of the American Urological Association, American Society for Radiation Oncology, and Society of Urologic Oncology, which recommend treatment intensification for patients undergoing sRT when risk factors, such as elevated PSA levels, higher ISUP, advanced T-stage, and pelvic lymph node metastases, are present. These factors have also been significant predictors of FFBS in our analysis. Additionally, our findings may help identify patients most likely to encounter biochemical failure by weighing risk factors against each other. Thus, our RSF model may allow more differentiated decision-making in terms of potential treatment intensification such as the administration of ADT.

As expected, our best RSF model performed better on the training dataset than on the validation datasets. One could suspect some indication of overfitting to the data from the training set since there was a difference of –0.10 in prediction performances between the training and the external outlier validation datasets. However, our best RSF model still outperformed our previously published nomogram, as the former almost reached a threshold of 0.70 regarding its performance in the external validation dataset. Due to the extensive recruitment in the study, and even if unbalanced classification setting, we deemed training and internal validation datasets to have appropriate numbers of events and numbers of patients (200/708, 28.2% and 77/271, 28%, respectively) to feel confident in the performance estimates. In the external validation dataset, the 50 patients experienced 24 events and performances, which may need further confirmation, as discussed later.

### Strengths

This study relied on solid methodological foundations. First, being multicenter, this study captured clinically relevant information across the differences in care management and clinical practices from 13 centers in 5 countries. Second, our recruitment period can be considered relatively short. Even in a retrospective setting, it helped reduce the impact of follow-up and care support that were not standardized from one center to another, from one country to another. Third, our training and internal validation datasets contained large numbers of patients and were highly comparable. This helped us choose the best model on similar and naïve data owing to unseen data when training the ML model (internal validation dataset). Fourth, all variables exhibited a reasonable unbalance across their categories during the training, without class counting less than 20% of patients except for the ISUP grade 5 and the sRT dose to the prostatic fossa <66 Gy, with 17.8% and only 6.6% of concerned patients, respectively. Fifth, we designed a prediction model study of type 3 according to the Transparent Reporting of a Multivariable Prediction Model for Individual Prognosis or Diagnosis (TRIPOD) statement, with the most dissimilar center being available as a separate dataset for validation [27]. Sixth, to compare the prediction accuracies of our best ML-based RSF with our previously published nomogram, we used the same design, including seeds, splits, and datasets, as those exploited for the nomogram. This allowed us to compare the exact and meaningful metrics (Harrell C-index and Brier score) on the internal validation dataset, and more importantly, the external outlier validation dataset. Seventh, we used the same variables to develop our previously published nomogram and our best ML-based RSF. No new variable was added for the training of our ML-based as compared to the development of our previously published nomogram.

### Limitations

Nevertheless, this study is subject to many limitations previously reported with our nomogram [15]. First, this study had a large patient cohort, but its multicenter nature meant different treatment regimens. Therefore, to make the model transferable, we did not include the variable “center” in the analysis. Second, our analysis is subject to bias inherent in retrospective studies, highlighting the need for prospective trials. Continuous variables were recorded, and this may have limited our ability to make better prognostic assessments. Third, the external outlier validation cohort, the same one used for our previous nomogram, only had 50 patients, which could affect the generalizability of the RSF model [15]. So, further evaluation within another external center, or even in another country, may help obtain more patients, thus providing more accurate estimates for predictive performances and better delineating the ability to generalize. We would then present a type 4 analysis, which is the highest degree of development and validation of a prediction model according to the TRIPOD classification of prediction model studies until TRIPOD-AI is published [27,28]. In addition, no other models than RSFs were trained. This could be done in the following work. In particular, it could be interesting to develop models, such as gradient boosting, support vector, or Bayesian theories, based on other theoretical grounds than those from decision trees.

Fourth, missing data were handled by exclusion only. This led to 141 potential patients being useless for developing and validating our previously published nomogram and our best RSF presented here. Creating missing data could be explored to detect those missing at random, and a replacement strategy could then be put in place, at least for some patients, in sensitivity analyses for the training. Sabbagh et al [29] expressed the criticism that our previously published nomogram was based on a Cox proportional hazards model without accounting for competing risks. One can address the same remark to our best RSF here. However, adapting ML algorithms in the presence of competing risks is still under development and is not yet fully ready for use. One application could be misleading in its interpretation and give a false conclusion.

### Future Directions

Despite these limitations, the findings of this study provide valuable insights into the possibility of integrating the RSF model when evaluating variables for predictive models, and the reliable performance of the RSF model in both validation sets enhances its applicability in real-world clinical settings. This includes the assessment of the personalized risk of FFBF, which could, in turn, lead to customized follow-up management or the assessment of risk stratification [27].

By going one step further, we are already aware that PSMA dosage and FFBF risk stratification are expected to expand in the next few months and years. This should influence patients’ management, follow-up, and prognosis by changing the probability of persistence or relapse. This means that our best RSF should be updated by retraining and transfer learning in some days, even if we cannot give a precise horizon yet. We also have developed a user-friendly app to facilitate easy access to our risk prediction model for clinicians and researchers [30].

### Conclusions

This study is the first prediction tool for PET-staged patients in the sRT field, highlighting the potential of an RSF model compared to a nomogram in predicting treatment outcomes. The RSF model demonstrated improved predictive accuracy compared to the model for identifying patients who may benefit from PSMA-PET-based sRT, maintaining robustness and generalizability across validation sets. Including additional variables in the RSF model, such as PSA persistence and pelvic local recurrence on PSMA-PET scans, provided valuable insights. Despite limitations, this study enhances the applicability of the RSF model in real-world clinical settings. It can improve patient outcomes and assist clinicians in making treatment decisions.

## Appendix

Multimedia Appendix 1 Participating centers and STROBE (Strengthening the Reporting of Observational Studies in Epidemiology) checklist.

## Abbreviations

ADT: androgen deprivation therapy
BR: biochemical recurrence
C-index: concordance index
FFBF: freedom from biochemical failure
ISUP: International Society of Urological Pathology
ML: machine learning
PSA: prostate-specific antigen
PSMA-PET: prostate-specific membrane antigen-positron emission tomography
pT stage: pathological T stage
RP: radical prostatectomy
RSF: random survival forest
sRT: salvage radiation therapy
STROBE: Strengthening the Reporting of Observational Studies in Epidemiology
TRIPOD: Transparent Reporting of a Multivariable Prediction Model for Individual Prognosis or Diagnosis

## Data Availability

The datasets generated or analyzed during this study are not publicly available due to ethical and legal issues regarding the General Data Protection Regulation but are available from the corresponding author upon reasonable request.
